# P-1878. Discontinuation of Clinical Studies on Infectious Diseases: A Cross-Sectional Analysis

**DOI:** 10.1093/ofid/ofaf695.2047

**Published:** 2026-01-11

**Authors:** Yousef R Alnomani

**Affiliations:** Faculty of Medicine, Benha University, Benha, Al Qalyubiyah, Egypt

## Abstract

**Background:**

Clinical studies in infectious diseases are vulnerable to discontinuation, representing significant sources of research waste in clinical medicine. This study evaluates the characteristics of clinical studies involving patients with infectious diseases to identify the reasons behind discontinuation to prevent this in the future.

**Figure:**
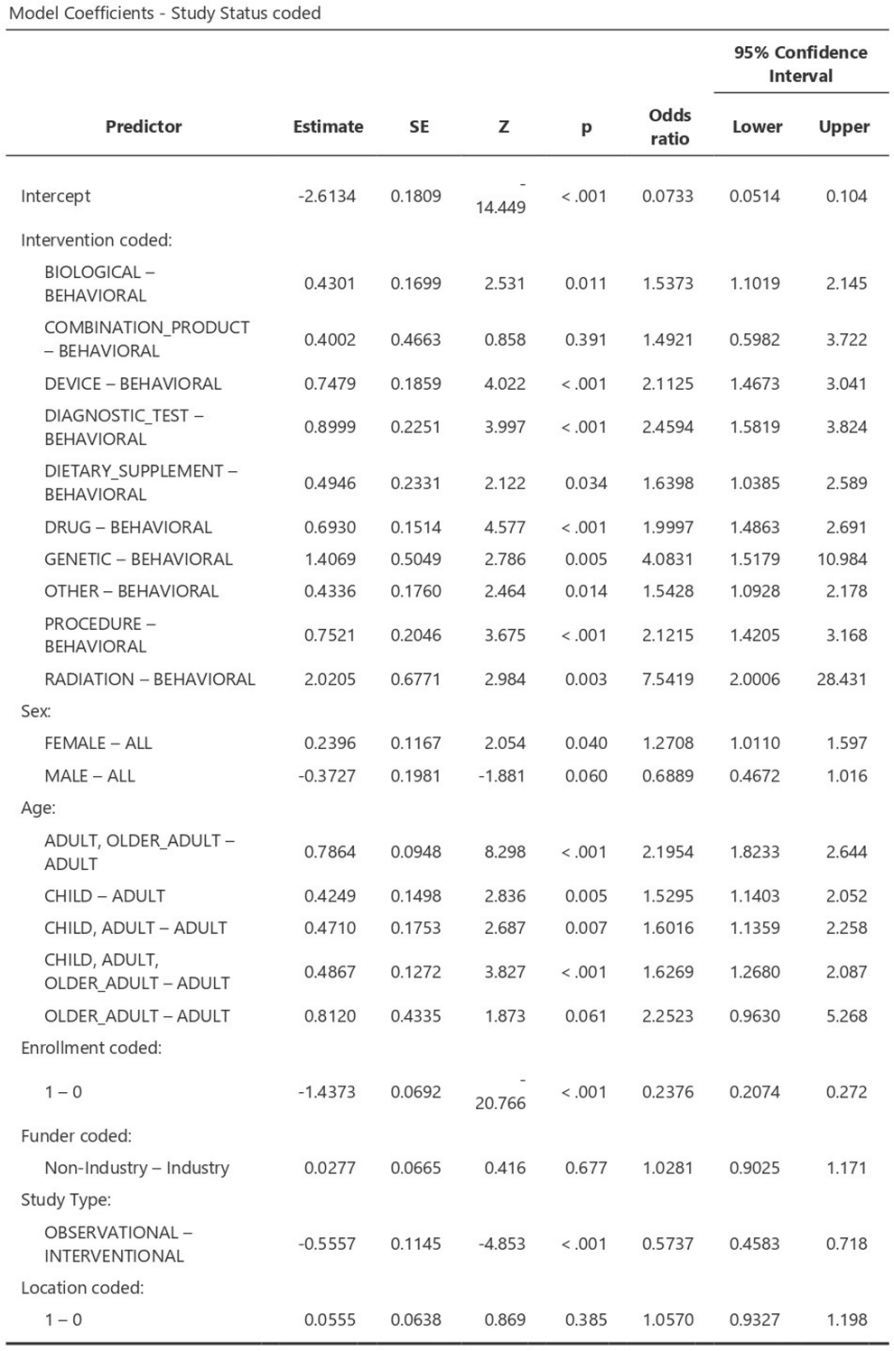
Binomial Logistic Regression Analysis Table

**Methods:**

We searched ClinicalTrials.gov for all clinical studies related to infectious diseases registered until April 2025. Studies completed after April 2023 and ongoing studies were excluded, as they may still be undergoing peer review. Data on enrollment, Gender, and intervention type were extracted and analyzed using binomial logistic regression analysis.

**Results:**

A total of 15,403 eligible registered clinical studies were included in the analysis. Of these, 13,365 (86.8%) were completed, and 2,038 (13.2%) were discontinued. The unadjusted logistic regression analysis identified several significant predictors:

- Studies with a small sample size (less than 100) were significantly more likely to be discontinued (OR = 0.24, 95% CI [0.21–0.27], *P* < 0.001).
- Studies that included only females were more prone to discontinuation (OR = 1.27, 95% CI [1.01–1.6], *P* = 0.04).
- Studies involving radiation had 7.5 times higher odds of discontinuation (OR = 7.54, 95% CI [2.00–28.43], *P* = 0.003).
- Studies on genetics had 4 times higher odds of discontinuation (OR = 4.08, 95% CI [1.5–10.99], *P* = 0.005).
- Diagnostic studies had 2.5 times higher odds of discontinuation (OR = 2.46, 95% CI [1.58–3.82], *P* < 0.001).

**Conclusion:**

There is evidence of non-dissemination bias in clinical studies of infectious diseases. This bias raises ethical concerns about exposing volunteer participants to potential risks without advancing medical knowledge.

**Disclosures:**

All Authors: No reported disclosures

